# Are foxes (*Vulpes* spp.) good sentinel species for *Toxoplasma gondii* in northern Canada?

**DOI:** 10.1186/s13071-022-05229-3

**Published:** 2022-04-01

**Authors:** Émilie Bouchard, Rajnish Sharma, Adrián Hernández-Ortiz, Kayla Buhler, Batol Al-Adhami, Chunlei Su, Heather Fenton, Géraldine G.-Gouin, James D. Roth, Chloé Warret Rodrigues, Carla Pamak, Audrey Simon, Nicholas Bachand, Patrick Leighton, Emily Jenkins

**Affiliations:** 1 Department of Veterinary Microbiology, University of Saskatchewan, Saskatoon, SK Canada; 2 Centre for Food-Borne and Animal Parasitology, Saskatoon, SK Canada; 3 Department of Microbiology, University of Tennessee, Knoxville, TN USA; 4 Ross University School of Veterinary Medicine, Basseterre, Saint Kitts and Nevis; 5 Nunavik Research Centre, Kuujjuaq, QC Canada; 6 Department of Biological Sciences, University of Manitoba, Winnipeg, MB Canada; 7 Nunatsiavut Research Centre, Nain, NL Canada; 8 Research Group On Epidemiology of Zoonoses and Public Health (GREZOSP), Université de Montréal, Saint-Hyacinthe, QC Canada

**Keywords:** *Toxoplasma gondii*, ELISA, MC-qPCR, Foxes, Sentinel species, Canada

## Abstract

**Background:**

In changing northern ecosystems, understanding the mechanisms of transmission of zoonotic pathogens, including the coccidian parasite *Toxoplasma gondii*, is essential to protect the health of vulnerable animals and humans. As high-level predators and scavengers, foxes represent a potentially sensitive indicator of the circulation of *T. gondii* in environments where humans co-exist. The objectives of our research were to compare serological and molecular assays to detect *T. gondii*, generate baseline data on *T. gondii* antibody and tissue prevalence in foxes in northern Canada, and compare regional seroprevalence in foxes with that in people from recently published surveys across northern Canada.

**Methods:**

Fox carcasses (*Vulpes vulpes*/*Vulpes lagopus*, *n* = 749) were collected by local trappers from the eastern (Labrador and Québec) and western Canadian Arctic (northern Manitoba, Nunavut, and the Northwest Territories) during the winters of 2015–2019. Antibodies in heart fluid were detected using a commercial enzyme-linked immunosorbent assay. *Toxoplasma gondii* DNA was detected in hearts and brains using a magnetic capture DNA extraction and real-time PCR assay.

**Results:**

Antibodies against *T. gondii* and DNA were detected in 36% and 27% of foxes, respectively. Detection of antibodies was higher in older (64%) compared to younger foxes (22%). More males (36%) than females (31%) were positive for antibodies to *T. gondii*. Tissue prevalence in foxes from western Nunavik (51%) was higher than in eastern Nunavik (19%). At the Canadian scale, *T. gondii* exposure was lower in western Inuit regions (13%) compared to eastern Inuit regions (39%), possibly because of regional differences in fox diet and/or environment. Exposure to *T. gondii* decreased at higher latitude and in foxes having moderate to little fat. Higher mean infection intensity was observed in Arctic foxes compared to red foxes. Fox and human seroprevalence showed similar trends across Inuit regions of Canada, but were less correlated in the eastern sub-Arctic, which may reflect regional differences in human dietary preferences.

**Conclusions:**

Our study sheds new light on the current status of *T. gondii* in foxes in northern Canada and shows that foxes serve as a good sentinel species for environmental circulation and, in some regions, human exposure to this parasite in the Arctic.

**Graphical Abstract:**

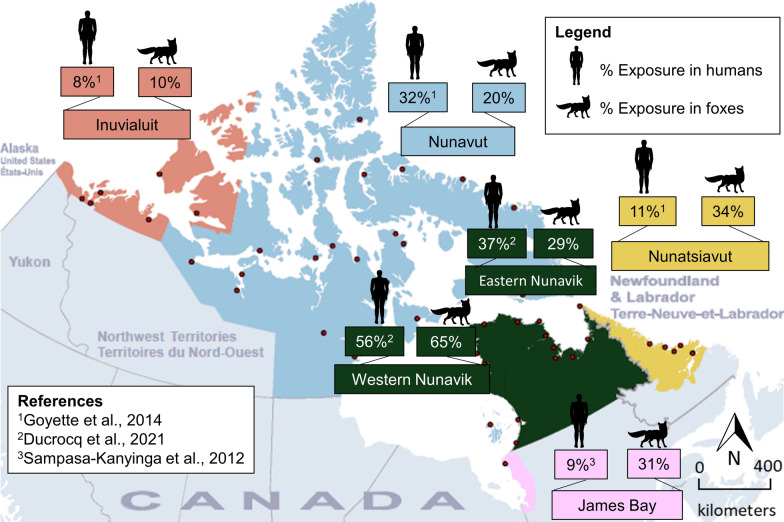

**Supplementary Information:**

The online version contains supplementary material available at 10.1186/s13071-022-05229-3.

## Background

The apicomplexan parasite *Toxoplasma gondii* is found in a vast array of ecosystems and can infect virtually all warm-blooded vertebrates. As approximately one third of the global human population has been exposed, *T. gondii* is one of the most successful parasites in the world [1, 2]. This parasite has multiple routes of transmission: environmental (contamination of soil, fresh produce, or water with sporulated oocysts shed in the feces of feline definitive hosts), meat-borne (through raw or undercooked meat containing bradyzoites), and vertical (mother to fetus via tachyzoites) [3]. While the infection may cause only mild symptoms and clinical signs, neurological, ocular, and reproductive problems may occur, especially if the immune system is compromised or in developing fetuses.

In humans, a higher seroprevalence is found in Latin America, parts of Eastern/Central Europe, the Middle East, parts of southeast Asia, and Africa [4]. This prevalence is probably due to greater levels of oocysts in the environment, where the definitive felid hosts are abundant and the climate is favorable, but could also be explained by cultural, hygienic, and nutritional habits that can influence levels of human exposure [4]. Lower seroprevalence has been observed in many European countries as well as the USA [4]. In Canada’s Arctic and subarctic regions, antibodies to *T. gondii* have been reported in people, with seroprevalence ranging from 8% in the western Arctic [5] to 40–60% in the eastern Arctic [6–8].

Globally, exposure to the parasite in human populations increases along a north-to-south gradient, but seroprevalence in some Inuit communities in Canada’s North is much higher than in other parts of North America. In Nunavik, northern QC, exposure to *T. gondii* varies regionally from 27 to 56% [9], significantly higher than the North American average of 10–20% [10, 11]. Wild felids are rarely seen above the treeline, and domestic cats are not a traditional companion animal in northern communities, although they have been brought from the south into communities [12]. Nevertheless, Inuit and wild carnivores (such as foxes) are likely exposed through handling and consumption of Arctic wildlife or from environmental sources, such as untreated freshwater [7]. Congenital toxoplasmosis has been reported in Nunavik, and seroconversion in pregnant women was significantly related to carcass processing and consumption of wild game (caribou meat) [13]. Game meat plays an important role in the cultural and traditional values of communities in the Canadian North and is an essential part in their well-being and food security. Seroprevalence of *T. gondii* has been reported in various northern wildlife species, including birds, ungulates, and carnivores [6, 14]; wild carnivores have thus the potential to act as sentinel animal hosts for food-borne parasites like *T. gondii*, sharing transmission dynamics with humans.

Animals can act as ideal sentinel hosts for select pathogens if they possess the following features: adequate availability (population stability), measurable response (e.g. parasites in tissues, antibodies in blood), earlier response than sympatric wild species or humans, high levels of exposure, and ideally do not serve as a direct source of human exposure [15, 16]. Arctic and red foxes are widespread across northern Canada and can be exposed to both oocysts shed into the environment by felids and tissue cysts in ingested meat and organs. Skinned, intact fox carcasses are easily accessible through local hunter and trapper organizations. Fox are not currently of conservation concern in Canada, and despite risks through handling of carcasses, they do not represent a risk of food-borne pathogens to people. In addition, high exposure to *T. gondii* has also been documented in foxes in some parts of the Canadian Arctic. For example, Bachand et al. [17] reported an exposure of 41% in 39 Nunavik foxes, quite similar to the regional prevalence of 42% (CI_95%_: 40–44) in people from this region [8]. Similarly, Bouchard et al. [18] found a seroprevalence of 39% (CI_95%_: 28–50) in Nunavut foxes compared to an exposure of 32% (CI_95%_: 29–36) in Inuit populations from Nunavut [5]. All these criteria make foxes good candidates to serve as sentinels for *T. gondii* circulation in Arctic ecosystems [17].

The current study has a much broader geographic scale and larger sample size than these previous surveillance studies, allowing, for the first time, a high level view of the suitability of foxes as indicators of environmental transmission [17, 18]. In addition, we capitalize on the relatively recent publications of human seroprevalence data from all Canadian Inuit regions from the International Polar Year Inuit Health Survey in 2007–2008 [5] and the Nunavik Health Survey in 2017 [9], as well as a study in Cree communities of James Bay [19], allowing comparison of trends in fox and human seroprevalence.

Although the fox population in Canada appears to be stable, elsewhere in the world, Arctic foxes (*Vulpes lagopus*) are affected by environmental change, such as increased competition with other carnivores like the red fox (*Vulpes vulpes*) moving onto the tundra [20, 21]. With climatic changes and anthropogenic activities affecting the Arctic at higher rates than anywhere else on the planet [22], this will alter the distribution and abundance of predators, prey, and parasites, including *T. gondii* and its hosts. Warmer, wetter, and more extreme climatic events (with the largest increases in temperature occurring in the western Canadian Arctic) will likely increase exposure rates to *T. gondii* in the Canadian North [6, 23]. Systematic surveillance for climate-sensitive pathogens such as *T. gondii* in suitable sentinel species, like foxes, will also allow us to detect changing risks due to climate change.

The methods used to screen for *T. gondii* can be indirect or direct. Serological tests are indirect, non-invasive methods commonly used in wildlife studies. They are rapid and simple to perform, but only give evidence of previous exposure through detection of antibodies, which for *T. gondii* appear to be long lived [24]. Less commonly, infection can be detected by using direct methods such as bioassays, detection of the parasite in tissues using immunohistochemical methods, or detection of parasite DNA by PCR [25]. Bioassays are gold standard tests to detect *T. gondii*, but they are expensive and time-consuming and sacrifice many animals (mice or cats) [26]. Commercially available kits extract DNA from only a small quantity of tissue (25–100 mg) and thus could easily miss tissue cysts that are randomly distributed and may occur at low densities [27]. Magnetic capture-qPCR (MC-qPCR) has higher sensitivity than DNA extraction kits due to its capacity to analyze up to 100 g of tissue [28] and has been successfully used in wildlife [17, 29]. Moreover, molecular methods are useful to further analyze the genotypes of *T. gondii* circulating in wild mammals. In North American wildlife, type 12 strain is considered to be a common genotype [30, 31], followed by types II and III, which are clonal lineages more commonly detected in livestock and people. As well, type II appears widespread in wildlife in the circumpolar region, which may reflect spillover from domestic cycles to wildlife and transport from sub-Arctic regions via migratory birds or in freshwater run-off [32]. Molecular epidemiology of *T. gondii* can reveal regional transmission dynamics, spatial patterns of *T. gondii* in relation to the genetic structure of hosts, and differences in virulence in animals and people [31, 33–36].

As serological tests are rarely optimized for wildlife, our first objective was to determine cutoff values for two serological tests (ELISA and IFAT), followed by comparison of these serological results with detection of DNA of the parasite in tissues using MC-qPCR. Our second objective was to determine antibody and tissue prevalence of *T. gondii* and associated risk factors in harvested foxes across northern Canada. Finally, our third objective was to compare regional seroprevalence for antibodies to *T. gondii* in harvested foxes (current study) and people (other studies) from different regions in northern Canada to determine if foxes are good sentinels of environmental transmission and human risk of exposure to this zoonotic parasite.

## Methods

### Sample locations

**Fig. 1:**
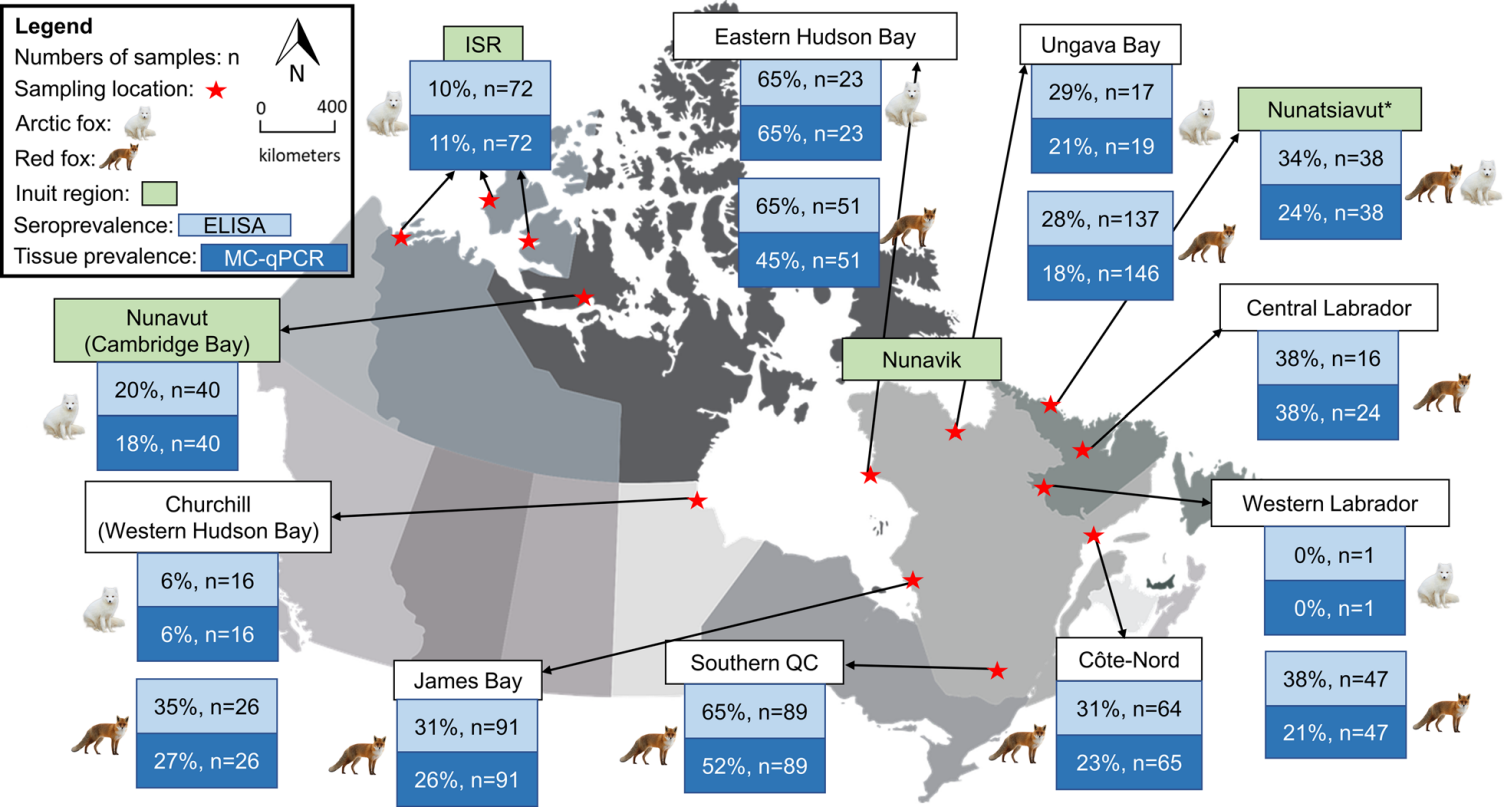
Prevalence of *T. gondii* and locations of foxes (*Vulpes vulpes/V. lagopus*, *n* = 749) harvested by local trappers in Canada: ISR (NT), Cambridge Bay (NU), Churchill (MB), Nunavik (QC), James Bay (QC), Côte-Nord (QC), Southern QC, Nunatsiavut (NL), Western Labrador (NL), and Central Labrador (NL). The asterisk (*) indicates that both *Vulpes* species were combined due to high number of unknowns

Harvested foxes were sampled from across northern Canada (> 4000 km), including all four Inuit regions of Canada: Inuvialuit Settlement Region (ISR) (Inuvik, Sachs Harbour, and Ulukhaktok, NT, Canada), Nunavut (Cambridge Bay, NU, Canada), Nunavik (QC), and Nunatsiavut (NL) (Fig. 1). These four regions and sampling sites are part of Canada’s North Coast region and are characterized by long, cold winters interrupted by short, cool summers. The western and northern parts of the Canadian Arctic coastlines, including regions of ISR and Nunavut, receive limited precipitation (< 300 mm annually) and experience relatively few storms. In contrast, the eastern Arctic, including Labrador/Nunatsiavut and Nunavik, experience much higher annual precipitation (up to 1000 mm) due to more frequent storms [37]. Samples were also collected in sub-Arctic regions including northern Manitoba (Churchill, MB), James Bay and Côte-Nord in QC, southern QC, and western and central Labrador (Fig. 1).

### Fox sampling

Foxes (red: *n* = 548, Arctic: *n* = 171, unknown: *n* = 30) were harvested by local trappers and collaborators during regular, licensed fur-trapping activities during the winters of 2015–2019. Carcasses were kept frozen at − 20 °C (in a freezer) or left outside in an enclosed shelter during winter (generally − 20 °C or colder), until shipped for necropsy at the Department of Environment and Natural Resources in Inuvik (NT), the Western College of Veterinary Medicine in Saskatoon (SK), the Churchill Northern Studies Centre (MB), the Faculté de Médecine Vétérinaire in Saint-Hyacinthe (QC), the Nunavik Research Center in Kuujjuaq (QC), and the wildlife division office at the Department of Fisheries, Forestry and Agriculture in Goose Bay (NL). As predilection sites for *T. gondii* in animals [38–40], whole hearts and brains were placed individually in identified plastic bags for each fox.

Species, sex, age, body condition, and location were recorded for most individuals. If the exact harvest coordinates were missing, coordinates from the closest community were used. Animals were classified as an “unknown *Vulpes* species” if they were from a location with known overlap in red and Arctic fox distribution and the species of the skinned carcass was not recorded. Age was determined based on tooth condition as per Chevallier et al. [41] or by counting cementum annuli (Matson’s laboratory, Manhattan, MT, USA). Foxes were classified as young (≤ 1 year old), mature (2–4 years old), and old (≥ 5 years old). An approximate body condition index (BCI) of fat deposits was rated visually using a scale of 1–3, as follows: (i) no to very little visceral fat deposits in the abdominal and peritoneal cavity; (ii) moderate visceral fat deposits; (iii) abundant visceral fat deposits.

### Serological analysis

#### Collection of heart fluid (HF)

As serum samples are difficult to obtain from harvested wildlife, we used fluid from thawed hearts, which has proven to be a good serum surrogate in which to detect antibodies to *T. gondii* [17, 29]. Despite long and suboptimal storage in remote regions, freezing and thawing should not have compromised the detection of antibodies in meat juice [42]. Hearts were frozen in Ziploc bags, then thawed, and tissue fluid from each bag was transferred to a 15 ml centrifuge tube using a sterile disposable plastic pipette and centrifuged at 3500*g* for 5 min. One milliliter was transferred in a labeled 1.5-ml Eppendorf tube, centrifuged again at 1000*g* for 5 min at 4 °C, and stored at 4 °C for use within the next 3 days or at − 20 °C for longer storage.

#### Determination of the optimal cutoff values for serological tests

As commercially available ELISA and IFAT assays were not validated in foxes, we evaluated sample dilutions for ELISA and IFAT. Samples from nine foxes known to be positive and negative on magnetic capture were used to determine cutoff values: three negative animals on MC-qPCR (Group N), three low positives (Cq value > 30; Group LP), and three high positives (Cq value ≤ 30; Group HP). The ELISA was performed at four dilutions: no dilution (ND), 1:2 (recommended as per manufacturer’s instruction), 1:4, and 1:8. The IFAT was performed at four dilutions (1:2, 1:10, 1:50, and 1:100), and the results were graded from 1 + to 3 + based on fluorescence intensity. The slides were screened by two blinded readers, and the results were compared. The between-reader repeatability of the IFAT was assessed using a weighted kappa value (*k*). Kappa values of ≤ 0.40, 0.40–0.60, 0.61–0.80, and ≥ 0.81 represented poor to fair, fair to moderate, moderate to substantial, and substantial to almost perfect agreement, respectively [43]. All samples were tested in duplicate. The selected optimal cutoff value for IFAT was at 1:50 dilution versus 1:2 for the ELISA.

#### Enzyme-linked immunosorbent assay (ELISA)

Antibodies to *T. gondii* were detected in heart fluid (1:2 dilution) using the commercially available ID Screen® Toxoplasmosis Indirect Multi-species kit (IDvet, Grabels, France). The ELISA was performed as per manufacturer’s instructions. The optical density (OD) was recorded at 450 nm using an ELISA microplate reader (Varioskan LUX multimode, ThermoFisher Scientific). Samples presenting an S/P % ≤ 40% were considered negative. Samples ≥ 70% were considered positive. If the S/P % was between 40 and 70%, the test result was considered doubtful and repeated. All samples were tested in duplicate. Positive and negative controls provided by the manufacturers were used in each batch. In addition, positive and negative serum samples from naturally exposed foxes were used as reference controls [18]. To check for cross-reactivity with other coccidian species, reference-positive serum samples for *Neospora caninum* (bovine) and *Hammondia hammondi* (feline) were provided by the Centre for Food-borne and Animal Parasitology (CFAP) in Saskatoon. The ELISA relative sensitivity and specificity using magnetic capture PCR as a reference test were 94% and 100%, respectively [29].

#### Indirect fluorescent antibody test (IFAT)

The IFAT was performed on heart fluid (1:50 dilution) using the commercially available antigen-coated Teflon-masked slides from VMRD following manufacturer’s instructions (VMRD, Pullman, WA, USA). Anti-canine IgG antibodies conjugated to fluorescein isothiocyanate (FITC; rabbit origin) were applied on slides. Slides were viewed under an Olympus DP70 fluorescence microscope at 40× objective. A complete staining around the tachyzoites was considered positive for *T. gondii* antibodies. Tachyzoites with only apical or no staining were recorded as negative. If discontinuous peripheral staining on tachyzoites was observed, the result was considered doubtful and repeated. All samples were tested in duplicate. Positive and negative serum samples from naturally exposed foxes were used as reference controls, previously confirmed by IFAT [18]. To check for cross-reactivity with other coccidian species, reference-positive serum samples for *N. caninum * (bovine) and *H. hammondi* (feline) were provided by the Centre for Food-borne and Animal Parasitology (CFAP) in Saskatoon. The IFAT relative sensitivity and specificity using magnetic capture PCR as a reference test were 94% and 100%, respectively [29].

#### Comparison of ELISA and IFAT

To determine the best-performing serological test, heart fluid from 158 randomly chosen foxes (117 red foxes, 29 Arctic foxes, 9 unknown) were tested (in duplicate) by ELISA and IFAT, and results were compared using magnetic capture PCR as a reference test.

#### Comparison of human and fox exposure

We used data from the International Polar Year Inuit Health Survey in 2007–2008, the Nunavik Health Survey in 2017, and Sampasa-Kanyinga et al. [19] to compare exposure of *T. gondii* in Inuit from Inuvialuit (NT), Nunavut (NU), Nunavik (QC), Nunatsiavut (NL), and Cree from James Bay (QC) to overall exposure in harvested foxes from the same regions. The human surveys used commercial (AxSYM, Abbott Diagnostics, Abbott Park, IL) and homemade immunoenzymatic assays (ELISAs) to detect IgG antibodies against *T. gondii* at the National Reference Centre for Parasitology (Montréal, QC, Canada) for Inuit communities and the National Microbiology Laboratory (Winnipeg, MB, Canada) for Cree communities.

### Molecular analyses

#### Magnetic capture: DNA extraction

Magnetic capture (MC)-qPCR was used to determine infection status and quantify infection intensity (parasite burden) based on detection of DNA of *T. gondii* [29, 32]. DNA was extracted from whole heart and brain combined as per Opsteegh et al. [28]. Each run included two spiked beef samples (positive controls) and one beef sample without spiking (negative control). The concentration of the undiluted *T. gondii* tachyzoite-stock (VEG type III) used for spiking was 2.5 × 10^6^/ml. A 10-times dilution series was made in ultrapure water to obtain 2.5 × 10^5^ and 2.5 × 10^4^/ml. For positive controls, 100 µl of these dilutions was added to 50 g of beef samples, resulting in samples spiked with 2500 and 25,000 tachyzoites. Cell-cultured tachyzoites of *T. gondii* were obtained from CFAP, Saskatoon. For each sample, a back-up of tissue lysate (50 ml) was kept at − 20 °C. The extracted DNA was stored at 4 °C if used in the following 3 days or at − 20 °C until further use.

#### Real-time PCR: DNA amplification and quantification

A real-time PCR using the Tox 9F (5′-aggagagata tcaggactgtag-3′) and Tox 11R (5′-gcgtcgtctc gtctagatcg-3′) primers for the detection of the 188 bp T*. gondii* sequence within the 529 repeat-element was performed using the Bio-Rad CFX 96 DNA thermal cycler (Biorad, Hercules, CA, USA), as per Bachand et al. [17]. A reaction was considered positive if (i) the Cq value was ≤ 35, (ii) the two positive extraction controls were positive, and (iii) the negative and two no-template controls were negative. Reactions with Cq values between 35 and 40 were considered positive if a 188-bp band was identified on gel electrophoresis. If only one of the two duplicates amplified, or if the CIAC amplification failed to occur, the PCR was repeated. Parasites were quantified using the following formula, log10 (tachyzoites) = (43.3 – Cq)/3.07), and expressed as number of tachyzoite equivalents (TEs) [32]. The intensity of infection was calculated by dividing TE by weight of the tissue processed and expressed as tachyzoite equivalents per gram (TEG).

#### Genotyping: DNA extraction and multiplex multilocus nested PCR–RFLP (Mn-PCR–RFLP)

Retained frozen lysate (50 ml) from strongly positive samples on MC-qPCR (*n* = 113, Cq value < 30) was thawed at room temperature, centrifuged at 3500 rpm for 15 min, and 250 µl transferred into a 1.5-ml Eppendeorf tube. DNA was extracted using the High Pure PCR Template Preparation kit (Roche, Mannheim, Germany). Protocol followed manufacturer’s instructions except the first step was skipped, as the lysate already contained proteinase K, and 75 µl was used for elution in the final step to increase DNA amount. All samples were extracted in duplicate and treated with 1 µl of RNAse A Solution (4 mg/ml, Promega, Madison, WI, USA). Frozen aliquots of template DNA (50 µl) were sent within 24 h to the Department of Microbiology, University of Tennessee, Knoxville, TN, USA, for further genetic characterization. A Mn-PCR–RFLP method employing ten genetic markers (SAG1, SAG2, SAG3, BTUB, GRA6, c22-8, c29-2, L358, PK1, and Apico [44]) was used as per Su et al. [35].

### Statistical analyses

#### Serological and molecular test agreement

Proportion of positive results was compared between the ELISA and IFAT, IFAT and MC-qPCR, and ELISA and MC-qPCR, using McNemar’s chi-square tests for paired data. If not significantly different, the kappa coefficient (*k*) was used to determine the level of agreement between the two tests. After being retested, all doubtful results from serological tests were considered negative. Analyses were performed using IBM SPSS (version 26; Armonk, New York, USA).

#### Association between quantitative MC-qPCR and ELISA results

A Spearman's rank-order correlation coefficient was performed to determine the covariation between antibody concentration (S/P%) and Cq values obtained from ELISA and MC-qPCR, respectively.

#### Prevalence and risk factors

Seroprevalence, tissue prevalence, and their 95% confidence intervals (CI) were calculated from the proportion of positive results using EpiTools epidemiological calculators [45]. We used a logistic regression with package lme4 v.1.1-26 [46] in R v.3.6.3 [47] to evaluate the effect of species, sex, age, latitude, and BCI on *T. gondii* prevalence (exposure and infection). Foxes with missing data were not included in the regression, nor were individuals of unknown species. We checked that no group was under-represented and that groups were distributed similarly in each category. We checked for multicollinearity between explanatory variables by using the variance inflation factor (VIF) (see Additional file 1: Text S1, Table S1, Fig. S1). Conditions affecting the prevalence of *T. gondii* in the fox population (e.g. climate-related factors, fox density, lemming density) may vary yearly. However, we could not include year as a fixed effect in our model as it was dependent on region (and thus latitude). We first produced a mixed-effect model including year as a random effect [48] and compared it to a corresponding fixed-effect model simply ignoring year. The outputs were very similar and did not change the main conclusion, and the intra-class correlation (ICC) for year as a random effect was low (ICC = 0.05). We thus selected the simpler fixed-effect model.

#### Infection intensity and risk factors

To evaluate the effect of species, sex, age, latitude, and BCI on the infection intensity of *T. gondii*, we used a generalized linear model (linear regression) in IBM SPSS (version 26; Armonk, New York, USA). We found no outliers (Cook’s distance was < 0.5) and no heteroskedasticity [49]. Infection intensity of *T. gondii* (tachyzoite equivalents per gram) was not normally distributed, and data were thus log 10 transformed (negative foxes were not included in the analysis). Foxes with missing data were also excluded from the model. An omnibus test was performed to evaluate the model coefficients (see Additional file 2: Text S1, Table S1a, b).

## Results

### Samples

Overall, seroprevalence was 36% (CI_95%_: 32–39) and tissue prevalence was 27% (CI_95%_: 24–31). Prevalence and number of samples per species, sex, age, geographic location, and BCI are shown in Table 1.

**Table 1:** Prevalence and risk factors in foxes (*Vulpes* spp; *n* = 749) harvested from Canada

		Seroprevalence^a^ (CI_95%_)/*N*	Tissue prevalence (CI_95%_)/*N*
Species	Red fox	41% (36–45)/530	30% (26–34)/548
	Arctic fox	21% (16–28)/169	20% (15–27)/171
	Unknown species	31% (17–49)/29	23% (12–41)/30
Sex	Male	36% (32–41)/387	27% (23–32)/397
	Female	31% (26–37)/293	25% (20–30)/302
	Unknown sex	60% (46–73)/48	44% (31–58)/50
Age	Young (≤ 1 year old)	22% (18–27)/370	19% (16–24)/383
	Mature (2–4 year old)	37% (30–44)/180	24% (18–30)/187
	Older (≥ 5 year old)	64% (52–74)/66	45% (34–57)/66
	Unknown age	62% (52–70)/112	50% (41–59)/113
Region	Northern Labrador, NL	34% (21–50)/38	24% (13–39)/38
	Central Labrador, NL	38% (18–61)/16	38% (21–57)/24
	Western Labrador, NL	38% (25–52)/48	21% (12–34)/48
	Ungava Bay, QC	29% (22–36)/154	19% (14–25)/165
	Eastern Hudson Bay, QC	65% (54–75)/74	51% (40–62)/75
	Côte-Nord, QC	31% (21–43)/64	23% (15–35)/65
	James Bay, QC	31% (22–41)/91	26% (18–36)/91
	Southern QC	65% (55–74)/89	52% (41–62)/89
	Churchill, MB	24% (13–39)/42	19% (10–33)/42
	Cambridge Bay, NU	20% (11–35)/40	18% (9–32)/40
	Ulukhaktok, NT	10% (5–19)/72	11% (6–20)/72
BCI	1 (no to very little fat)	32% (26–38)/250	25% (20–30)/256
	2 (moderate fat)	28% (23–34)/293	21% (16–25)/297
	3 (abundant fat)	42% (32–52)/91	30% (22–40)/92
	Unknown BCI	64% (54–73)/94	51% (41–60)/104
Total		36% (32–39)/728	27% (24–31)/749

*N* number of individuals tested, *CI* confidence intervals, *BCI* body condition index

^a^Individuals not tested (no heart fluid) = 21

### Specificity of serological assays

No cross-reactivity was observed on positive serum samples for *H. hammondi* or *N. caninum* antibodies using ELISA or IFAT.

### Cutoff values and choice of serological assay

#### Evaluation of sample dilutions for ELISA and IFAT

Discordance was observed between dilutions of both ELISA and IFAT and the three groups of foxes on MC-qPCR for only two animals: dilution 1:2 on IFAT for a fox in Group N (ID BJ2016-223) and one in Group LP (ID W2015-218) (Table 2). Since the IFAT results were discordant for dilution 1:2, dilution 1:50 was used for further analyses, as per Bouchard et al. [18]. Since the four dilutions on ELISA gave similar results for all individuals, we used the cutoff value of 1:2 as recommended by the manufacturer. One MC-qPCR positive fox (ID W2015-218) showed negative results on both serological tests (except dilution 1:2 for IFAT), suggesting that antibody levels had not yet been reached or had declined below detection limits post-exposure/infection. The results are presented in Table 2.

**Table 2:** Comparison of *Toxoplasma gondii* results for heart fluid samples using serological methods (ELISA and IFAT) relative to magnetic capture real-time PCR to determine the best dilution for cutoff values

Dilutions	ID	ELISA S/P%				IFAT			
		ND	1:2	1:4	1:8	1:2	1:10	1:50	1:100
N	BJ2016-223	27.31	13.84	13.3	11.56	1 +	N	N	N
	W2015-221	13.3	2.33	1.68	1.19	N	N	N	N
	Mi2015-214	21.34	2.99	1.47	1.36	N	N	N	N
LP	We2015-248	243.81	237.62	227.31	195.87	3 +	3 +	2 +	1 +
	We2015-253	344.84	325.35	296.58	281.05	3 +	3 +	2 +	2 +
	W2015-218	17.92	8.31	6.13	2.5	1 +	N	N	N
HP	We2015-250	351.79	332.19	323.13	295.77	3 +	3 +	3 +	3 +
	W2015-227	297.94	272.58	239.47	203.53	3 +	3 +	2 +	1 +
	FN21	331.65	309.55	291.8	244.46	3 +	3 +	2 +	2 +

Positive results: ELISA ≥ 70% and IFAT ≥ 1 +

*N* negative, *LP* low positive on magnetic capture (Cp value > 30), *HP* high positive on magnetic capture (Cp value ≤ 30), *ND* no dilution, *ELISA* enzyme-linked immunosorbent assay, *IFAT* indirect fluorescent antibody test

#### Comparison of serological methods (IFAT and ELISA) relative to MC-qPCR

Antibodies to *T. gondii* were detected in 53% (83/158) of foxes using ELISA and in 46% (72/158) using IFAT. There was excellent agreement for the between-reader repeatability of the IFAT (*k* = 0.88). There was no statistical difference between serological and molecular results using the McNemar chi-square test (ELISA: χ^2^ = 0.643, *df* = 1, *p* = 0.423, *n* = 158; IFAT: χ^2^ = 1.161, *df* = 1, *p* = 0.281, *n* = 158). There was substantial agreement for ELISA (*k* = 0.82) and only moderate agreement for IFAT (*k* = 0.61) compared to MC-qPCR. The relative sensitivity and specificity of ELISA were 94% and 89%, respectively, compared to 76% and 85% for IFAT. ELISA was therefore used for subsequent analyses.

### Final agreement between ELISA and MC-qPCR

Seventy foxes had positive serology and negative molecular tests, nine were serologically negative but tissue positive, 190 were positive on both, and 459 were negative on both. There was no statistical difference between serological and molecular results using the McNemar chi-square test (χ^2^ = 45.6, *df* = 1, *p* < 0.001, *n* = 728). There was substantial agreement between the two tests (*k* = 0.75).

### Association between quantitative MC-qPCR and ELISA results

A moderate, negative, statistically significant correlation was found between ELISA antibody concentration (S/P%) and Cq values on MC-qPCR (Spearman’s correlation coefficient = − 0.419, *p* < 0.001). As Cq values are inversely related to DNA quantity, this means higher antibody levels were associated with higher amounts of DNA.

### Detection of *T. gondii* antibodies and associated risk factors

Antibodies to *T. gondii* were detected in 36% (CI_95%_: 32–39) of foxes using ELISA (heart fluid was missing for 21 individuals). Seropositivity increased from young (22%, CI_95%_: 18–27), to mature (37%, CI_95%_: 30–44), to older (64%, CI_95%_: 52–74) foxes. More males (36%, CI_95%_: 32–41) than females (31%, CI_95%_: 26–36) were positive for antibodies to *T. gondii*. In general, seroprevalence was highest in foxes in eastern Hudson Bay and southern QC and lowest in the western Canadian Arctic (MB, NT, western NU). Finally, antibodies were detected more frequently in foxes with abundant fat (42%, CI_95%_: 32–52) than moderate (28%, CI_95%_: 23–34) and little fat (32%, CI_95%_: 26–38). The results are presented in Table 1 and Fig. 1.

Our logistic regression included 583 complete observations. Our final model for seroprevalence included sex, age, latitude, and BCI as significant risk factors. The odds of presence of *T. gondii* antibodies were three times (odds ratio: 2.65, CI_95%_: 1.11–6.92, *p* = 0.036) higher in male than female foxes. Mature and old foxes had significantly higher exposure to *T. gondii* than young foxes—two times (odds ratio: 1.99, CI_95%_: 1.31–3.02, *p* = 0.001) and seven times (odds ratio: 6.86, CI_95%_: 3.74–12.96, *p* < 0.001), respectively. The probability of being exposed to *T. gondii* decreased at higher latitude as well as for foxes having moderate to little fat (Table 3).

**Table 3:** Coefficients of the final logisitic regression model and risk factors associated to *Toxoplasma gondii* serological and tissue prevalence in foxes (*Vulpes* spp.) in Canada

Variables	β (SE)	*p* value	*df*	OR (CI_95%_)
Variables for seroprevalence				
Species, red fox	0.42 (0.50)	0.404	1	1.52 (0.59–4.28)
Sex, male	0.97 (0.46)	0.036*	1	2.65 (1.11–6.92)
Age, mature	0.69 (0.21)	0.001*	2	1.99 (1.31–3.02)
Age, old	1.93 (0.32)	< 0.001*	2	6.86 (3.74–12.96)
Latitude	− 0.07 (0.02)	< 0.001*	1	0.93 (0.88–0.98)
BCI, low	− 0.60 (0.30)	0.042*	2	0.55 (0.31–0.98)
BCI, medium	− 0.66 (0.29)	0.024*	2	0.52 (0.29–0.92)
Species^^^sex, male red fox	− 0.73 (0.51)	0.156	1	0.48 (0.17–1.29)
Variables for tissue prevalence				
Species, red fox	0.14 (0.59)	0.807	1	1.15 (0.39–4.02)
Sex, male	1.48 (0.53)	0.005*	1	4.39 (1.66–13.88)
Age, mature	0.36 (0.24)	0.137	2	1.43 (0.89–2.29)
Age, old	1.13 (0.33)	< 0.001*	2	3.10 (1.62–5.86)
Latitude	− 0.08 (0.03)	0.003*	1	0.92 (0.87–0.97)
BCI, low	− 0.48 (0.32)	0.139	2	0.62 (0.33–1.18)
BCI, medium	− 0.66 (0.33)	0.044*	2	0.52 (0.28–0.99)
Species^^^sex, male red fox	− 1.19 (0.59)	0.042*	1	0.30 (0.09–0.91)

*BCI* body condition index, *Species*^*^*^*Sex* interaction term, *β* estimate coefficient, *SE* standard error, *df* degree of freedom, *OR* odds ratio, *CI*_*95%*_ 95% confidence interval

^*^Statistically significant at *p* < 0.05

**Fig. 2:**
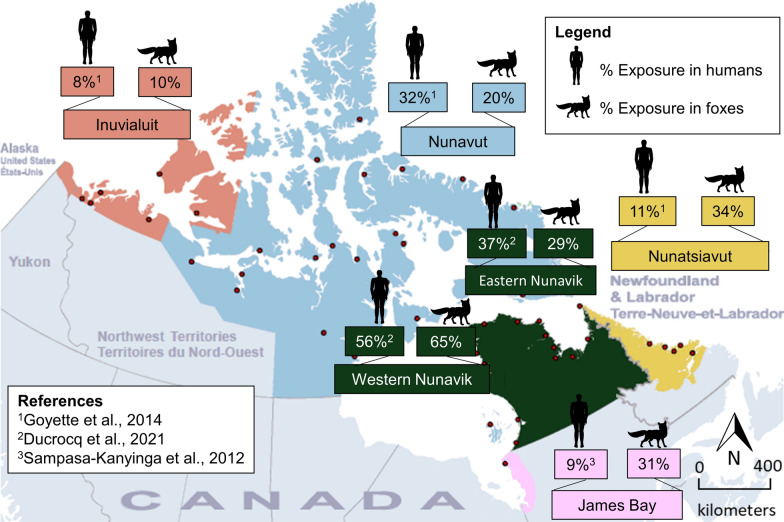
Exposure of *T. gondii* in people and foxes harvested in Inuvialuit (NT), Nunavut (NU), Nunavik (QC), Nunatsiavut (NL), and James Bay (QC), Canada. Red dots indicate northern communities. Results from both fox species were combined. Credit map: Statistics Canada

According to previous studies [5, 9, 19], exposure of *T. gondii* in Inuit was 8% (*n* = 362, CI_95%_: 4–12) in the Inuvialuit region, 32% (*n* = 1923, CI_95%_: 29–36) in Nunavut, 37% (*n* = 2735, CI_95%_: 33–40) in eastern Nunavik, 56% (*n* = 3532, CI_95%_: 52–61) in western Nunavik, 11% (*n* = 310, CI_95%_: 6–16) in Nunatsiavut, and 9% (*n* = 266, CI_95%_: 6–13) in James Bay. Regional human seroprevalences were compared to regional fox seroprevalences in the present study in Fig. 2.

### Detection of *T. gondii* DNA and associated risk factors

DNA of *T. gondii* was detected in 27% (CI_95%_: 24–31) of foxes using MC-qPCR. Older foxes (45%, CI_95%_: 34–57) were more frequently infected than mature (24%, CI_95%_: 18–30) and young (19%, CI_95%_: 16–24) foxes. A trend in location similar to seroprevalence was also observed for detection of *T. gondii* DNA in foxes. The results are presented in Table 1 and Fig. 1.

Our logistic regression included 593 complete observations and revealed that sex, age, latitude, BCI, and interaction of sex and species were significantly associated with the presence of *T. gondii* DNA. The odds of presence of *T. gondii* DNA were four times (odds ratio: 4.39, CI_95%_: 1.66–13.88, *p* = 0.005) higher in male than female foxes. Old foxes had three times higher odds of being positive for DNA of *T. gondii* in tissues (odds ratio: 3.10, CI_95%_: 1.62–5.86, *p* < 0.001) than young foxes. The probability of being infected with *T. gondii* decreased at higher latitude as well as for male red foxes and foxes having moderate fat (Table 3).

### Infection intensity of *T. gondii* and associated risk factors

Mean infection intensity was 8879 TEG (CI_95%_: 4278–13,480). Mean (SE and CI_95%_) TEG among different classes of risk factors is shown in Table 4. Our generalized linear model included 123 positive foxes with complete data and revealed that interaction between BCI and latitude was significantly associated with infection intensity. Coefficients of the generalized linear models and potential risk factors for infection intensity (log transformed) of *T. gondii* in foxes are provided in Table 5.

**Table 4:** Infection intensity (tachyzoites equivalent per gram) of *Toxoplasma gondii* in foxes (*Vulpes* spp*.*) in Canada

Variables	Category (*N*)	Mean TEG (CI_95%_); SE
Species	Arctic foxes (30)	27,117.46 (5939.70–48,295.21); 10,354.71
	Red foxes (137)	5315.85 (1824.39–8807.31); 1765.54
Age	Young (67)	13,137.21 (2938.79–23,335.84); 5108.04
	Mature (40)	6755.22 (− 817.35–14,327.79); 3743.81
	Old age (24)	4154.19 (− 2916.23–11,224.62); 3417.88
Sex	Male (98)	11,210.20 (3794.33–18,626.07); 3736.48
	Female (58)	6319.44 (576.25–12,062.62); 2868.06
BCI	High (24)	5609.90 (− 3793.16–15,012.96); 4545.49
	Medium (51)	15,687.50 (3030.87–28,344.14); 6301.34
	Low (56)	5632.37 (− 596.50–11,861.25); 3108.15

*BCI* body condition index, *N* number of foxes in the respective category for which TEG was estimated (TEG was not estimated for all positive tissues on MC-qPCR because of lack of data, e.g. missing heart or brain), *TEG* tachyzoite equivalents per gram, *CI*_*95%*_ 95% confidence interval, *SE* standard error

**Table 5:** Coefficients of the generalized linear model and potential risk factors for infection intensity (tachyzoites equivalent per gram of tissues; log transformed) of *Toxoplasma gondii* in foxes (*Vulpes* spp.) in Canada

Variables	β (SE)	95% Wald confidence interval		Hypothesis test		
		Lower	Upper	Wald-chi-Square	*df*	*p*-value
First model						
Species, Arctic fox	0.228 (0.3744)	− 0.506	0.962	0.371	1	0.542
Sex, male	0.209 (0.2568)	− 0.294	0.713	0.664	1	0.415
BCI, low	0.113 (0.3259)	− 0.526	0.751	0.119	1	0.730
BCI, medium	0.581 (0.3327)	− 0.071	1.233	3.046	1	0.081
Latitude	0.020 (0.0302)	− 0.039	0.079	0.448	1	0.503
Age, young	0.384 (0.3200)	− 0.244	1.011	1.438	1	0.231
Age, mature	0.320 (0.3377)	− 0.342	0.982	0.898	1	0.343
Final model						
BCI^*^*^lat, low BCI	0.033 (0.0212)	− 0.008	0.075	2.478	1	0.115
BCI^*^*^lat, medium BCI	0.042 (0.0207)	0.002	0.083	4.152	1	0.042*
BCI^*^*^lat, high BCI	0.030 (0.0216)	− 0.013	0.072	1.895	1	0.169

*BCI* body condition index, *β* estimate coefficient, *SE* standard error, *df* degree of freedom, *BCI*^*^*^*lat* interaction term

^*^Statistically significant at *p* < 0.05

### Genotyping results

Out of 113 fox samples with Cq value < 30, no samples amplified following multiplex multilocus nested PCR-RFLP.

## Discussion

This is the first large-scale, nationwide study on *T. gondii* in a naturally infected wild animal species, using validated serological and molecular testing. The scope of this work allowed geographic comparisons that are not possible when individual or regional studies are conducted using different methods and over different time frames. It also represents a spatio-temporal baseline against which future changes in prevalence driven by climate change can be compared. Finally, we compared prevalence and distribution of *T. gondii* in foxes in four Inuit regions with human exposure data collected through the International Polar Year Inuit Health Survey in 2007–2008 and the Nunavik Health Survey in 2017 [5, 9], as well as James Bay Cree [19], and found that foxes were remarkably good sentinels for human seroprevalence in most regions (Fig. 2).

After determining optimal dilutions and conducting our pilot study on heart fluid, we detected better agreement with MC-qPCR using ELISA compared to IFAT. In contrast, Sharma et al. [29] reported excellent agreement between both ELISA and IFAT for antibodies to *T. gondii* in wolverines (*Gulo gulo*), using MC-qPCR as a reference test. IFAT has subjective endpoint criteria based on visual inspection, which can lead to bias when reporting seroprevalence. ELISA results are quantitative and therefore more objective and had higher relative sensitivity and specificity than IFAT in the current study. For these reasons, we used ELISA as our primary serological test.

We detected antibodies to *T. gondii* in 36% of the foxes using ELISA, whereas we detected DNA of *T. gondii* in 27% of foxes. Sixty-seven foxes were positive on ELISA but negative on MC-qPCR. This was not unexpected, as seroprevalence is often higher than tissue prevalence [31, 50, 51]. This discrepancy can be explained by the presence of cysts in tissues other than brain and heart or by a lower tissue-infection intensity than the detection limit of the technique [52]. On the other hand, nine foxes were positive on MC-qPCR and negative on serology, possibly due to acute infection (where tachyzoites or early tissue cysts are present but antibodies have not yet been produced at detectable levels) or senescent infections (where antibody levels have declined, with tissue cysts persisting in a non-immunogenic state) [28, 53]. Even though the agreement between ELISA and MC-qPCR was substantial (*k* = 0.75), using both serological and molecular tests concurrently maximized detection of *T. gondii* in foxes in the current study.

The overall detection of antibodies was higher in red foxes compared to Arctic foxes, but this was not significant when both species were present at the same latitude. The species effect in our model is likely hidden by the latitude effect (see Additional file 1: Fig. S1). As most red foxes were trapped below the tree line, higher prevalence was expected because of the presence of sympatric wild felids, such as lynx, or outdoor domestic cats that could contribute to environmental contamination and higher infection prevalence in prey species of foxes. In contrast to the southern bias in prevalence, a higher mean infection intensity (tachyzoites equivalent per gram) was observed in Arctic foxes compared to red foxes. Higher parasitic intensity in Arctic foxes could be due to a higher infection intensity of *T. gondii* in food sources of Arctic foxes, as they may be less exposed to oocysts, and possibly due to higher level of carnivorous specialization in the Arctic versus red fox [54]. As well, Arctic foxes could be more susceptible to infection than red foxes, or there could be a more transmissible genotype in northern Canada [55]. Further studies are warranted to explore these hypotheses. Also, we noted that older foxes had significantly higher seroprevalence and tissue prevalence than young foxes. Older age is often associated with *T. gondii* exposure [56, 57], probably because the likelihood of exposure increases with age, and both tissue cysts and antibodies are long-lived [58]. However, mean infection intensity was more than double in young compared to mature and old foxes, which could be due to lower immunity in young animals, allowing more parasite replication in tissues, as either tachyzoites or bradyzoites.

Higher seroprevalence and tissue prevalence were observed in foxes from Hudson Bay (65% and 51%) versus Ungava Bay (29% and 19%) in Nunavik, northern QC. This difference was unexpected, as lynx (potential sources of *T. gondii*) are present in Ungava Bay, but not Hudson Bay. A similar pattern is seen in people, with a seroprevalence of 36% in Ungava Bay and 56% in Hudson Bay [8]. The seroprevalence in Inuit seems to increase with consumption of marine mammals (especially seal), fish, and birds [8]. According to the health survey ‘Nutrition and Food Consumption Among the Inuit of Nunavik’ [59], the consumption frequency of marine mammals was higher in Hudson Bay, whereas land animals were most frequently consumed in Ungava Bay. Carnivores in coastal regions, including the majority of the trapped foxes in our study, would also have access to fish and carcasses of marine mammals. Environmental contamination by oocysts may therefore not be a major source of exposure for foxes. Sources of infection with *T. gondii* for marine animals are thought to be oocysts washed into the sea through water run-off and transported by marine currents [34, 60–62]. Marine mammals could get infected by consuming filter feeding fish and invertebrates acting as mechanical reservoirs of oocysts [63, 64]. We also detected a higher seroprevalence and tissue prevalence in foxes in southern QC (65%) versus sub-Arctic QC (31%). This difference could be explained by higher densities of both wild and outdoor domestic cats in southern QC, thus increasing contamination of food and water with oocysts for both foxes and their prey.

Our overall geographical findings indicate widespread exposure to and infection with *T. gondii* in foxes across northern Canada; however, both fox and human seroprevalences were lower in the western Canadian Arctic, which is drier and may experience less run-off of oocysts from sub-Arctic regions [37]. For instance, western Hudson Bay had substantially lower sero- and tissue prevalence than eastern Hudson Bay, and the lowest fox and human seroprevalence was in the ISR in the western-most Canadian Arctic. This is also consistent with observation of low (3%) seroprevalence of antibodies to *T. gondii* in people in Alaska [65]. Prevalence in foxes also decreased as latitude increased, suggesting that colder and drier climate may affect the survival or the infectivity of *T. gondii* parasite in more northern regions. Our results in foxes were surprisingly well correlated with previous studies in Inuit populations, demonstrating a lower seroprevalence in ISR (8%) and Nunavut (32%) compared to the more southern region of Nunavik [5]. However, Inuit from Nunatsiavut and Cree from James Bay had a seroprevalence of 11% [5] and 9% [19], respectively, compared to 34% and 31% in foxes, possibly reflecting dietary preferences in Inuit in Nunatsiavut and James Bay Cree for cooked meat and/or consumption of terrestrial (versus marine) wildlife [7]. Indeed, Inuit from Nunavut and Nunavik consume high quantities of seal meat [8, 66], but not in ISR and Nunatsiavut, where *T. gondii* seroprevalence is much lower. Caribou meat is the most consumed country food in ISR and Nunatsiavut [67, 68], which would be expected to have low prevalence of *T. gondii* in tissues [69].

Determining the genotypes/strains of *T. gondii* present in foxes across the Canadian Arctic would provide valuable information about the population structure and transmission of this parasite; for example, if strains in foxes were identical to those in marine mammal species or felids in sub-Arctic regions. However, as we found, recovering sufficient DNA of *T. gondii* from naturally infected wildlife can be challenging, as they have low tissue burden and therefore yield low levels of DNA [70]. In future, minimizing freeze-thaw cycles and using larger amounts of tissue for extraction could maximize DNA yield. Future studies, including DNA characterization assays, are needed to determine genotypes of *T. gondii* circulating in foxes in northern Canada.

The effect of climate change on *T. gondii* ecology still remains uncertain in northern regions, but may be more severe in the western North American Arctic, where the parasite is not currently as successful as in the Eastern Arctic, and the magnitude of rapid and directional climate change is more pronounced than almost anywhere in the globe. The western Canadian Arctic (Yukon, Northwest Territories, and Nunavut) is currently experiencing the largest increases in warming and precipitation due to climate change, which could bring increases in survival and infectivity of oocysts in the future [23]. As a result, the range and abundance of *T. gondii* may expand via aquatic environments and migratory wildlife in these ecosystems. The presence of fish and marine invertebrate species in new areas that were previously too cool for their survival represents a risk for increased spread, since they can accumulate oocysts in their gills and filtration organs [71]. As well, with warmer climate, the distribution of lynx and their prey species is predicted to move northward with the tree line, facilitating local oocyst transmission [6]. Increased precipitation could also enhance survival and transport of oocysts from water sources flowing from south to north.

## Conclusions

This study supports foxes as appropriate sentinels for potential human exposure and transmission of *T. gondii* in a future of climate change. Foxes are widespread across northern Canada, year-round residents, and exposed at similar rates and routes as humans in most of the Canadian Arctic. While the western Arctic may be most vulnerable, the higher prevalence in the eastern Canadian Arctic warrants continued study to monitor prevalence of *T. gondii* in wildlife species that are consumed in northern communities. Future research could investigate additional predictors of *T. gondii* presence and prevalence in foxes, such as diet (terrestrial vs. marine food sources). The geographic scale of the present study speaks to significant community engagement from trappers and territorial, provincial, and indigenous governments. Synergizing sentinel animal surveillance with human disease surveillance is critical to identifying and addressing the potential human and animal health risks associated with altered transmission of *T. gondii* in a rapidly changing Arctic.

## Supplementary Information

**Additional file 1**: **Fig. S1**. Density of data for each species (Arctic fox in blue and red fox in yellow) compared to latitude in decimal coordinates indicating strong relationships (VIF = 146)

**Additional file 2: Table S1.** a Model selection results for hypotheses of risk factors influencing *Toxoplasma gondii* infection intensity in foxes in Canada. b Model selection results for hypotheses including “BCI^*^*^lat” interaction term and other possible risk factors influencing *Toxoplasma gondii* infection intensity in foxes in Canada.

## Data Availability

The datasets generated during and/or analysed during the current study are available from the corresponding author on reasonable request.

## Abbreviations

AIC: Akaike’s Information Criterion
BCI: Body condition index
CFAP: Centre for Food-borne and Animal Parasitology
CIAC: Competitive internal amplification control
ELISA: Enzyme-linked immunosorbent assay
IFAT: Indirect fluorescent antibody test
MC-qPCR: Magnetic capture and real-time PCR
PCR: Polymerase chain reaction
qPCR: Quantitative PCR
TEG: Tachyzoite equivalents per gram
VIF: Variance inflation factor
