# Correction to: Are foxes (*Vulpes* spp.) good sentinel species for *Toxoplasma gondii* in northern Canada?

**DOI:** 10.1186/s13071-022-05277-9

**Published:** 2022-04-25

**Authors:** Émilie Bouchard, Rajnish Sharma, Adrián Hernández-Ortiz, Kayla Buhler, Batol Al-Adhami, Chunlei Su, Heather Fenton, Géraldine-G. Gouin, James D. Roth, Chloé Warret Rodrigues, Carla Pamak, Audrey Simon, Nicholas Bachand, Patrick Leighton, Emily Jenkins

**Affiliations:** 1 Department of Veterinary Microbiology, University of Saskatchewan, Saskatoon, SK Canada; 2 Centre for Food-Borne and Animal Parasitology, Saskatoon, SK Canada; 3 Department of Microbiology, University of Tennessee, Knoxville, TN USA; 4 Ross University School of Veterinary Medicine, Basseterre, Saint Kitts and Nevis; 5 Nunavik Research Centre, Kuujjuaq, QC Canada; 6 Department of Biological Sciences, University of Manitoba, Winnipeg, MB Canada; 7 Nunatsiavut Research Centre, Nain, NL Canada; 8 Research Group on Epidemiology of Zoonoses and Public Health (GREZOSP), Université de Montréal, Saint-Hyacinthe, QC Canada

## Correction to: Parasites & Vectors (2022) 15:1–14 10.1186/s13071-022-05229-3

Following publication of the original article [1], the authors flagged the following to us:

“On pages 3 and 5 of the PDF, the following can be seen:

Sachs Harbour, and Ulukhaktok, NT, USA), Nunavut (Cambridge Bay, NU, USA)

and

the National Reference Centre for Parasitology (Montréal, QC, USA) for Inuit communities and the National Microbiology Laboratory (Winnipeg, MB, USA) for Cree communities.

However, these 2 regions and both national centres are in Canada, not in the USA.”

The published article has since been corrected, replacing ‘USA’ with ‘Canada’ where applicable. The publisher apologizes for this oversight.

As well, it was brought to the authors’ attention that one of the names in the author list had been provided with the incorrect spelling. Namely, ‘Géraldine-G. Gouin’ had been incorrectly spelled as ‘Géraldine G.-Gouin’. The error has since been corrected in the original article.
