# Supplementary material for: Are foxes (Vulpes spp.) good sentinel species for Toxoplasma gondii in northern Canada?
Source: Parasit Vectors. 2022 Apr 1;15:115. doi: 10.1186/s13071-022-05229-3 (PMC8972674; doi:10.1186/s13071-022-05229-3)
Supplement: Supplementary file 1 — Additional file 1: Fig. S1. Density of data for each species (Arctic fox in blue and red fox in yellow) compared to latitude in decimal coordinates indicating strong relationships (VIF = 146) [file 13071_2022_5229_MOESM1_ESM.docx]

**Supplementary information**

**Additional file 1: Text S1.** For serological and tissue prevalence, we tested the relevance of possible interaction terms by comparing models with Akaike information criterion (AIC), where models with AIC < 2 were considered equally plausible. Models were tested against the null model to see if there was a significative amelioration (Table S1). Values of the variance inflation factor (VIF) exceeded 10 for species^^^latitude interaction, indicating collinearity (Fig. S1). This term was therefore not included in the model. However, we elected to retain species since the species^^^sex interaction term was significant.

**Additional file 1: Table S1.** Akaike’s information criterion model selection results for hypotheses of risk factors influencing *Toxoplasma gondii* serological and tissue prevalence in foxes in Canada.

| **Model** | **Models for serological prevalence** | **∆AIC** | **AIC weight** | **Log likelihood** | **k** |
| --- | --- | --- | --- | --- | --- |
| 1 | ELISA ~ species^^^sex + lat + age + BCI | 0.00 | 0.36 | -324.98 | 9 |
| 2 | ELISA ~ species^^^BCI + sex+ lat + age | 0.30 | 0.31 | -324.13 | 10 |
| 3 | ELISA ~ species^^^BCI + lat + age + species^^^sex | 0.50 | 0.28 | -323.23 | 11 |
| 4 | ELISA ~ species^^^age + sex +lat + BCI | 3.72 | 0.06 | -325.84 | 10 |
| 5 | ELISA ~ species^^^sex^^^age + lat + BCI | 9.57 | 0.00 | -323.77 | 15 |
| 6 Null | ELISA ~ 1 | 56.34 | 0.00 | -361.15 | 1 |
|  | **Models for tissue prevalence** |  |  |  |  |
| 1 | MC ~ species^^^sex + lat + age + BCI | 0.00 | 0.64 | -275.27 | 9 |
| 2 | MC ~ species^^^BCI + lat + age + species^^^sex | 1.92 | 0.25 | -274.23 | 11 |
| 3 | MC ~ species^^^BCI + sex+ lat + age | 4.20 | 0.08 | -276.37 | 10 |
| 4 | MC ~ species^^^age + sex +lat + BCI | 6.18 | 0.03 | -277.36 | 10 |
| 5 | MC ~ species^^^sex^^^age + lat + BCI | 9.40 | 0.01 | -273.97 | 15 |
| 6 Null | MC ~ 1 | 20.10 | 0.00 | -293.32 | 1 |

As per lme4 notation, the ^^^ indicates that the model ran the fixed effect of each factor independently and the 2 and 3 ways interactions / AIC: Akaike’s information criterion / ΔAIC: change in AIC relative to top model / k: the number of model parameters / MC: Magnetic capture / ELISA: Enzyme-linked immunosorbent assay / lat: study site latitude / BCI: Body condition index


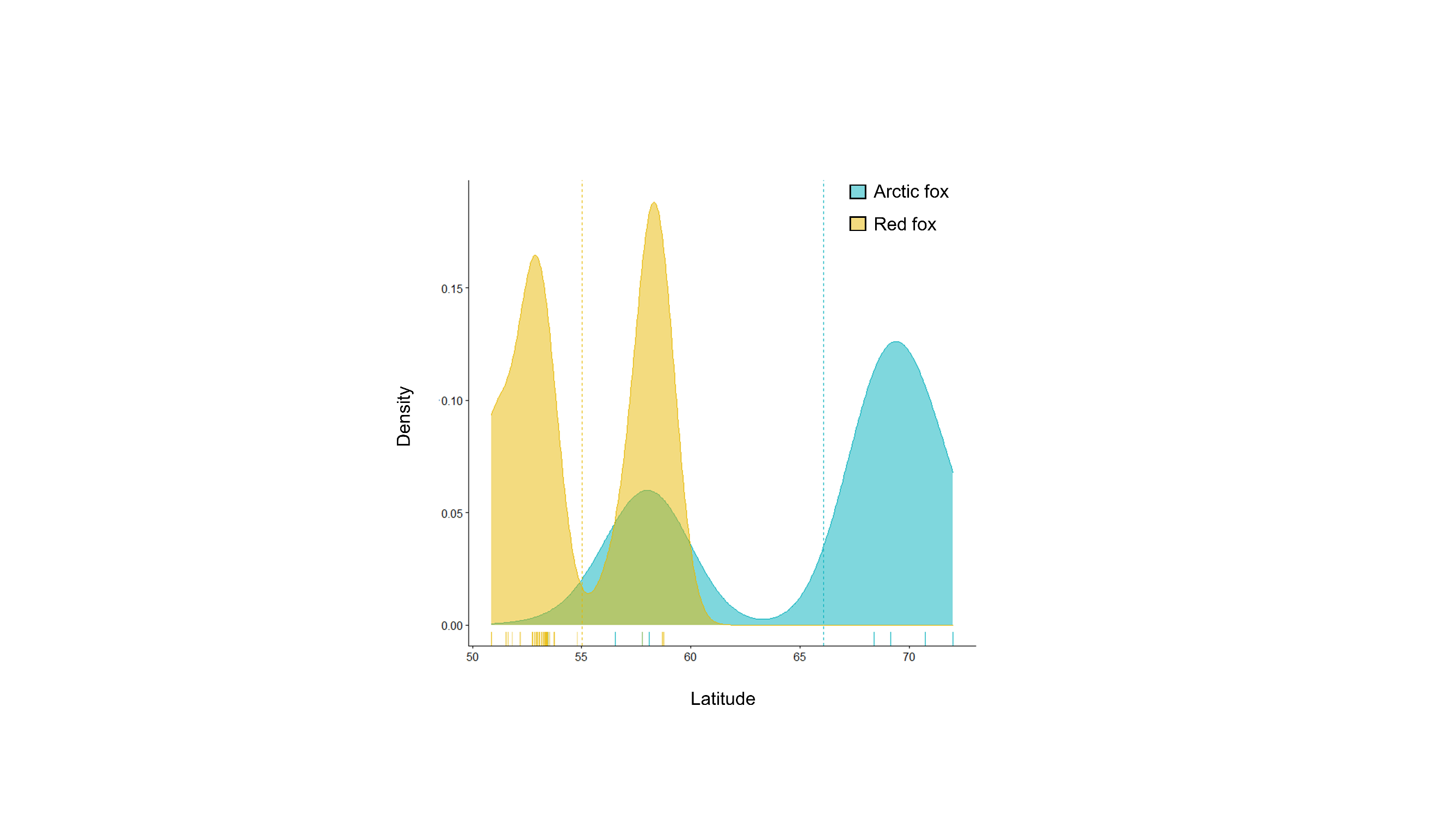


**Additional file 1: Figure S1**: Density of data for each species (Arctic fox in blue and red fox in yellow) compared to latitude in decimal coordinates indicating strong relationships (VIF=146).
