# Supplementary material for: Are foxes (Vulpes spp.) good sentinel species for Toxoplasma gondii in northern Canada?
Source: Parasit Vectors. 2022 Apr 1;15:115. doi: 10.1186/s13071-022-05229-3 (PMC8972674; doi:10.1186/s13071-022-05229-3)
Supplement: Supplementary file 2 — Additional file 2: Table S1. a Model selection results for hypotheses of risk factors influencing Toxoplasma gondii infection intensity in foxes in Canada. b Model selection results for hypotheses including “BCI^lat” interaction term and other possible risk factors influencing Toxoplasma gondii infection intensity in foxes in Canada. [file 13071_2022_5229_MOESM2_ESM.docx]

**Supplementary information**

**Additional file 2: Text S1.** For infection intensity, AIC was used for the selection of the final model; model (s) with lower AIC_C_ value (finite sample corrected AIC) was (were) considered better than the other models. Models with AIC < 2 were considered equally plausible. BCI and latitude were associated with infection intensity and interaction was observed between them; we therefore included BCI and latitude as interaction in the final model. Further models were tested for including BCI^^^lat interaction term along with other possible risk factors influencing *T. gondii* infection intensity in foxes in Canada. However, BCI^^^lat interaction was kept in the final model as aforementioned (Table S1 a-b).

**Additional file 2: Table S1 a).** Model selection results for hypotheses of risk factors influencing *Toxoplasma gondii* infection intensity in foxes in Canada.

| Model | Models for infection intensity | Δ AIC_C_ | AIC_C_ | Log Likelihood | Omnibus Test | | |
| --- | --- | --- | --- | --- | --- | --- | --- |
|  | | | | | **LR**  **Chi-sq** | **df** | **p** |
| 1 | ii ~ sex + species + age + BCI+ lat | 0 | 421.272 | -200.839 | 12.214 | 7 | 0.094 |
| 2 | ii ~ sex + age + BCI+ lat | 1.959 | 419.313 | -200.025 | 11.843 | 6 | 0.066 |
| 3 | ii ~ age + BCI+ lat | 1.706 | 417.607 | -201.317 | 11.260 | 5 | 0.046 |
| 4 | ii ~ BCI + lat | 2.434 | 415.173 | -202.330 | 9.233 | 3 | 0.026 |
| 5 | ii ~ BCI^^^lat | 0.943 | 414.230 | -201.859 | 10.176 | 3 | 0.017* |

**Additional file 2: Table S1 b).** Model selection results for hypotheses including “BCI^^^lat” interaction term and other possible risk factors influencing *Toxoplasma gondii* infection intensity in foxes in Canada.

| Model | Models for infection intensity | Δ AIC_C_ | AIC*_C_* | Log Likelihood | Omnibus Test | | |
| --- | --- | --- | --- | --- | --- | --- | --- |
|  |  |  |  |  | **LR Chi-sq** | **df** | **p** |
| 1 | ii ~ BCI^^^lat+ species + age + sex | 0 | 420.257 | -200.332 | 13.229 | 7 | 0.067 |
| 2 | ii ~ BCI^^^lat + age + sex | 1.968 | 418.289 | -200.292 | 12.865 | 6 | 0.045 |
| 3 | ii ~ BCI^^^lat + age | 1.676 | 416.613 | -200.819 | 12.254 | 5 | 0.031 |
| 4 | ii ~ BCI^^^lat | 2.383 | 414.230 | -201.859 | 10.176 | 3 | 0.017* |

ii: Infection intensity

lat: Study site latitude

BCI: Body condition index

BCI^^^lat: Interaction term

AIC_C:_ Finite sample Corrected Akaike’s Information Criterion

ΔAIC: change in AIC_C_ relative to top model

LR: Likelihood ratio

df: Degree of freedom

^*^Statistically significant at p<0.05
